# Allo-Reactivity of Mesenchymal Stem Cells in Rhesus Macaques Is Dose and Haplotype Dependent and Limits Durable Cell Engraftment *In Vivo*

**DOI:** 10.1371/journal.pone.0087238

**Published:** 2014-01-29

**Authors:** Iryna A. Isakova, Calvin Lanclos, Julie Bruhn, Marcelo J. Kuroda, Kate C. Baker, Veena Krishnappa, Donald G. Phinney

**Affiliations:** 1 Center for Bioenvironmental Research, Tulane University Health Sciences Center, New Orleans, Louisiana, United States of America; 2 Department of Immunology, Tulane National Primate Research Center, Covington, Louisiana, United States of America; 3 Department of Veterinary Medicine, Tulane National Primate Research Center, Covington, Louisiana, United States of America; 4 Kellogg School of Science and Technology, The Scripps Research Institute, Jupiter, Florida, United States of America; 5 Department of Molecular Therapeutics, The Scripps Research Institute, Jupiter, Florida, United States of America; Emory University School of Medicine, United States of America

## Abstract

The emerging paradigm that MSCs are immune privileged has fostered the use of “off-the-shelf” allogeneic MSC-based therapies in human clinical trials. However, this approach ignores studies in experimental animals wherein transplantation of MSCs across MHC boundaries elicits measurable allo-immune responses. To determine if MSCs are hypo-immunogeneic, we characterized the immune response in rhesus macaques following intracranial administration of allogeneic vs. autologous MSCs. This analysis revealed unambiguous evidence of productive allo-recognition based on expansion of NK, B and T cell subsets in peripheral blood and detection of allo-specific antibodies in animals administered allogeneic but not autologous MSCs. Moreover, the degree of MHC class I and II mismatch between the MSC donor and recipient significantly influenced the magnitude and nature of the allo-immune response. Consistent with these findings, real-time PCR analysis of brain tissue from female recipients administered varying doses of male, allogeneic MSCs revealed a significant inverse correlation between MSC engraftment levels and cell dose. Changes in post-transplant neutrophil and lymphocyte counts also correlated with dose and were predictive of overall MSC engraftment levels. However, secondary antigen challenge failed to elicit a measurable immune response in allogeneic recipients. Finally, extensive behavior testing of animals revealed no main effect of cell dose on motor skills, social development, or temperament. Collectively, these data indicate that allogeneic MSCs are weakly immunogenic when transplanted across MHC boundaries in rhesus macaques and this negatively impacts durable engraftment levels. Therefore the use of unrelated donor MSCs should be carefully evaluated in human patients.

## Introduction

Mesenchymal stem cells (MSCs) have demonstrated efficacy in treating inflammatory, ischemic, and immunological disorders in experimental animal models [1] and have yielded promising results in human clinical trials [2]. Over the past decade MSCs have emerged as potent regulators of adaptive and innate immune effector cells. For example, MSCs suppress T cell proliferation in response to allo-antigens [3], [4] and induce the formation of T cells with a regulatory phenotype [5]. They also inhibit the differentiation of naïve CD4 T cells into pro-inflammatory T_H_17 cells [6], block dendritic cell maturation and function [7], secrete factors that enhance neutrophil anti-microbial activity and chemotaxis [8] and suppress NK cell activation and cytolysis [9]. These findings have spurred the use of off-the-shelf allogeneic MSC-based therapies in humans despite the established role of major histocompatibility antigens in graft rejection.

In contrast, studies conducted in experimental animals indicate that allogeneic MSCs trigger donor-specific cellular and humeral immune responses *in vivo.* For example, pre-clinical studies conducted in rodents [10]–[13], swine [14], and non-human primates [15], [16] demonstrate that allogeneic MSCs induce measurable anti-donor T and B cell mediated responses. Indeed, the detection of donor-specific antibodies in the serum of transplant recipients provides clear evidence of allo-antigen recognition by B cells. These findings are consistent with reports indicating that allogeneic MSCs exhibit shorter retention times *in vivo* [17], [18] and accelerate rejection of solid organs [19]. Species specific differences in MSC allo-reactivity may result from the fact that allograft responses in humans are evaluated using mixed lymphocyte reactions *in vitro* [20]–[23], which fail to predict the ability of cells to suppress graft vs. host disease in human patients [23]. Therefore, the immune privileged status of allogeneic MSCs remains ambiguous and questions their utility for treating human disease.

Previously we conducted pre-clinical studies in infant rhesus macaques to evaluate the safety of direct intracranial MSC transplantation as a potential treatment for neurologic disorders [16], [24], [25]. These studies revealed that allogeneic MSCs transplanted to immuno-competent macaques exhibited durable but low engraftment throughout various anatomical structures along the brain neuraxis but also resulted in transient increases in lymphocyte counts in peripheral blood, which were cell dose dependent. Herein, we examined in more detail the immune response in macaques following intra-cranial administration of allogeneic vs. autologous MSCs, which revealed clear evidence of productive allo-recognition based on expansion of NK, B and T cell subsets in peripheral blood and detection of allo-specific antibodies in allogeneic transplant recipients. Moreover, the magnitude of the response was influenced by the degree of mismatch between the MSC donor and recipient. Consistent with these results, our analysis also revealed an inverse correlation between cell dose and MSC engraftment levels in brain at six months post-transplant. In addition, absolute changes in post-transplant neutrophil and lymphocyte counts in peripheral blood correlated with MSC dose and were predictive of overall MSC engraftment levels. Due to the close similarity between the immune systems of non-human primates and humans, results of these studies have important implications for the use of allogeneic MSCs in clinical medicine.

## Materials and Methods

### Study Subjects

Infantile female rhesus macaques (*Macaca mulatta)* were housed individually in standard infant cages, allowed social contact on a regular basis, and provided standard enrichment including manipulable items in the cage, various food supplements, task-oriented feeding methods and human interaction with caretakers and research staff. Enrichment was tailored to the species as dictated by the Animal Welfare Act and outlined in the Tulane National Primate Research Center Policy on Environmental Enrichment. Animals showing signs of psychological distress through behavior or appearance received special attention including additional enrichment devices, alterations to room configurations, and/or clinical intervention. Animals were maintained on standard diets and food restriction was not employed at any time as part of the study regimen. Animals were subjected to routine physical exams on a weekly basis by the veterinary staff during which time animal body temperature and weight were recorded. Animals were also routinely monitored for neurological impairments, such as paralysis or alterations in behavior that increased suceptibility to injury or caused pain and distress. All animals enrolled in the study exhibited normal weight gain compared to age match controls over the study time course and completed the study without experiencing adverse side effects. Medical care for all animals was provided by the veterinary staff and at no time during the study was such care restricted. Animals were euthanized by anesthesia with ketamine hydrochloride followed by overdose with sodium pentobarbital. All aspects of animal care and scientific evaluation of the macaques was conducted in accordance with institutional guidelines and approved by the Institutional Animal Care and Use Committee of Tulane University and The Scripps Research Institute and were compliant with guidelines established by the Association for Assessment and Accreditation of Laboratory Animal Care (AALAC), the United States Department of Agriculture (USDA) and Office of Laboratory Animal Welfare (OLAW). All animals tested negative for STLV, B-VIRUS, and SIV.

### Cell Isolation and Flow Cytometry

Allogeneic MSCs were isolated from the bone marrow of male rhesus macaques raised in the virus-free colony at the New England National Primate Research Center as described previously [25]. Animals at the New England Primate Research Center (NEPRC) were maintained in accordance with standards of the Association for Assessment and Accreditation of Laboratory Animal Care and the Harvard Medical School Animal Care and Use Committee. Animal experiments were approved by the Harvard Medical Area Standing Committee on Animals and conducted according to the principles described in the Guide for the Care and Use of Laboratory Animals. Autologous MSCs were isolated from bone marrow aspirates of infant macaques (3–4 weeks) housed at the Tulane National Primate Research Center under a protocol approved by Institutional Animal Care and Use Committee of Tulane University and The Scripps Research Institute. All samples were negative for foamy virus at time of harvest. MSCs were culture expanded for two passages prior to transplantation as described previously [25]. Peripheral blood (1.2 ml) was harvested from all transplant recipients and shams at approximately 2 and 1 month prior to surgery and at 10, 30, 60, 90, and 150 days post-surgery. PBMNCs were isolated via gradient centrifugation, washed 3× with PBS and stained with a panel of human antibodies that cross-react with rhesus epitopes CD2, CD3, CD4, CD8, CD16, CD20, NKp46, KIR, IL15aR, CD138, CCR7, CD25, CD56, and HLADR (BD Biosciences, San Jose, CA). Flow cytometry was performed using a Becton-Dickinson LSR II System (BD Biosciences) and complete blood cell counts were quantified using a Hematology Analyzer Advia 120 (Siemens, Malvern, PA).

### Surgical Procedures

Animals were subjected to routine physical examinations by the veterinary staff on a weekly basis before and after surgery. Infant macaques were administered MSCs at 6–8 weeks of age as described previously [25]. Each animal received four paired 25 µl injections (1.0 µl/min) of MSCs (12.5×10^3^ cells/µl) or PBS targeted to the caudate nucleus using coordinates determined from MRI scans of the brain. Six months later, each transplant recipient was administered a single dose (1×10^6^ cells) of donor MSCs via four subcuticular injections (4 mm apart) into the right thigh. Blood samples were drawn up to 14 days post-challenge after which time animals were sacrificed.

### Haplotype Analysis

Genomic DNA prepared from PBMNCs was used as input in PCR reactions containing primers specific to each of the following rhesus macaque alleles; A01, A02, A08, A11, B01, B03, B04, B08, B17, and DRBw201. RNA isolated from PBMNCs was transcribed into cDNA and amplified by PCR using multiple primers for the rhesus Mamu class I genes followed by pyro-sequencing as described [26].

### Cyto-toxicity Assays and Serum Immunoglobulin Levels

Reactivity of host PBMNCs against respective donor MSCs was evaluated using the Cyto-Tox 96® non-radioactive cyto-toxicity assay kit (Promega, Madison, Wisconsin, USA) according to the manufacturer’s instructions. Briefly, PBMNCs (1×10^5^ cells) were co-cultured in multi-well plates (0.32 cm^2^) with donor MSCs (1×10^4^ cells/well) for 4 hours at 37°C and then levels of cytosolic lactate dehydrogenase released into the media was quantified using a colorimetric assay. The percentage of cell-mediated toxicity was calculated using the formula provided by the manufacturer. Serum samples (400-fold dilution) obtained from clotted peripheral blood were analyzed using the Monkey IgG ELISA kit (Alpha Diagnostics Int., San Antonio, TX) according to the manufacturer instructions. All assays were performed in duplicate. Donor MSCs were also incubated for 1.5 h at 37°C in serum (50%) isolated from each respective transplant recipient and then stained with an antibody (1∶10 dilution) specific to the λ-chain of rhesus immuno-globulins (BD Biosciences) and analyzed by flow cytometry.

### Behavioral Tests

All macaques at 7 and 14 days of age were subjected to a 20-minute battery of tests adapted for non-human primates from the Brazelton Neonatal Behavioral Assessment Scale used with humans to document baseline motor function, temperament, and interactive skills [27]. The test evaluated visual orientation and attention span, state control, motor maturity, activity, reflexes and responses, fine and gross motor skills and strength, and temperamental items such as vocalization, self-quieting abilities, fearfulness, and distress. Test scores were grouped into four categories (orientation, control, motor maturity, activity) and compared to those obtained for 109 age-, sex-, and rearing-matched infants (Champoux, unpublished). Subjects were also placed in a socialization/exercise cage (or ‘play cage’) in groups of three to six individuals on a daily basis. Play cage data were examined at 3, 5, and 6 months of age. During each 5-minute observation, 13 aspects of behavior were quantified with a numerical scale in ascending order of maturity and activity. These items were grouped into four categories (large motor, small motor, social, behavioral state) for analysis. Each behavioral category was analyzed using repeated measures ANOVA. Finally, animals were also assessed post-surgically using the Modified Bayley test of infant development as described previously [25]. Twenty-one separate motor and behavioral test variables were collapsed into 3 scores for analysis (problem-solving, motor abilities, and temperament). All subjects were tested at 3 months post-surgery and again at 6 months post-surgery.

### Real-time PCR Analysis

MSC engraftment levels in brain were estimated by quantifying male DNA levels via real-time PCR as described previously [25]. PCR reactions (200 ng total DNA) containing the following primers and Taqman® probe: 5′-6-FAM-TGC AGT TTG CTT CCG GCA GAT CC-TAMRA-3′ (Life Technologies, Grand Island, NY); forward PCR primer, 5′-GGC GAA GAT GCT GCA AAA C-3′; reverse PCR primer 5′-TTC TCT GCA GGG TAC CGA AGA-3′ (Integrated DNA Technologies, Coralville, IA) were amplified using a 7900 HT sequence detector (Applied Biosystems, Carlsbad, CA). The slope of each amplification plot was determined by linear regression and the efficiency (e) of each amplification reaction was calculated as e = 10^(−1/slope)^/2*100. PCR reactions were run in quadruplicate and results averaged.

### Statistical Analysis

Differences between time points were evaluated using the students T test and values of p<0.05 were considered to be statistically significant. Statistical differences between treatment groups were evaluated using single factor or two-factor without replication analysis of variance (ANOVA) and correlations were evaluated by calculating the Pearson product moment correlation coefficient. Values with α = 0.05 and p<0.05 were considered statistically significant. Post-hoc analysis was performed using the Tukey-Kramer method due to the fact that group sizes varied between experimental groups. The critical range for the Tukey-Kramer method was calculated using the formula: q*SQRT((MS_W_/2*(1/n_1_+1/n_2_)) where q is the studentized range distribution, MS_W_ is the mean square within group, and n is the number of observations per group.

## Results

### Allogeneic MSC Administration Induces Expansion of PBMNCs in Immuno-competent Rhesus Macaques

**Figure 1:**
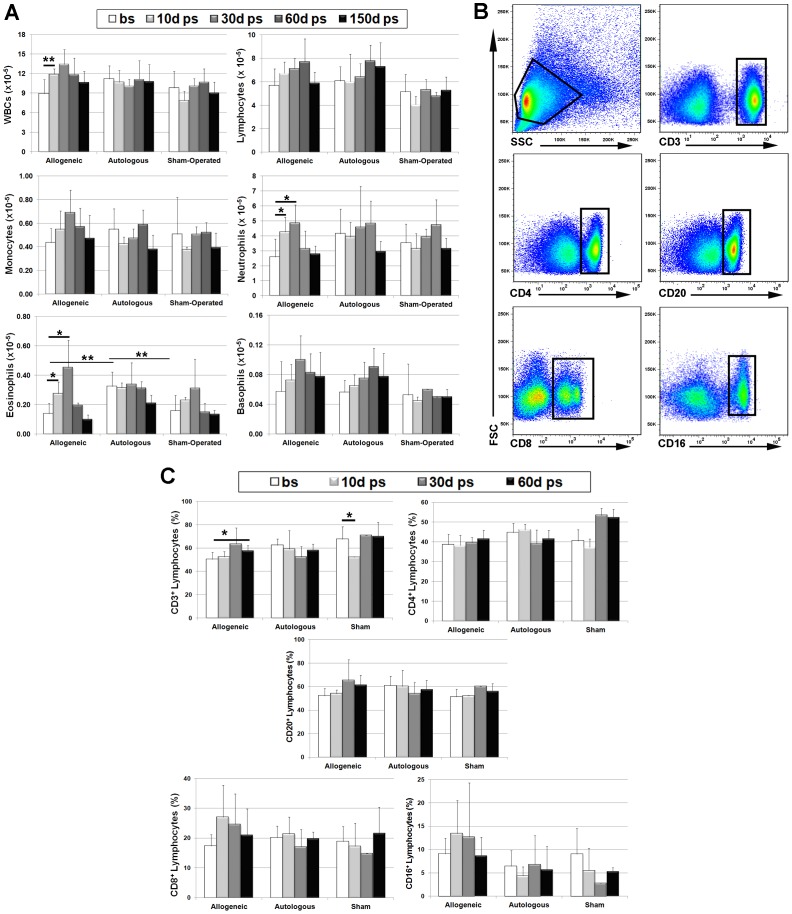
Administration of allogeneic but not autologous MSCs induces transient expansion of PBMNCs. (**A**) Peripheral blood samples harvested before surgery (bs) and at 10, 30, 60, and 150 days post-surgery (ps) were analyzed by automated counting to determine the number of WBCs, lymphocytes, monocytes, neutrophils, eosinophils and basophils. Plotted values (mean ± SD) represent cell counts averaged for all transplant recipients within each respective treatment group at the indicated time points. Cell counts taken at 1 month and 2 weeks before surgery were averaged to determine pre-surgical baseline values. (**B**) Representative density plots illustrating the gating strategy used to remove dead cells and doublets and identify lymphocyte subsets expressing CD3, CD4, CD20, CD8, and CD16 in peripheral blood by flow cytometry. (**C**) Plotted values (mean ± SD) represent circulating levels of each lymphocyte subset averaged for all transplant recipients within each respective treatment group at the indicated time points. * P<0.05, **P<0.01. P values were determined using unpaired, two-tailed Students t Test throughout.

To assess MSC allo-reactivity, we analyzed the systemic immune response of immuno-competent rhesus macaques following intracranial injection of 2.5×10^6^ autologous (n = 4) or allogeneic (n = 4) MSCs. Two animals administered an equal volume of PBS served as sham-operated controls. A transient but significant (p<0.05) increase in circulating WBC, neutrophil, and eosinophil counts was observed at 10 and/or 30 days post-transplant as compared to pre-surgical baseline levels only in the allogeneic treatment group (Fig. 1A). Significant differences [F(2,12) = 9.67, p = 0.003] in lymphocyte counts was also evident between groups. In these studies, pre-surgical blood cell counts were not significantly different between treatment groups except for eosinophils, which were significantly (p<0.01) higher in the autologous vs. the allogeneic and sham treatment groups.

We next used flow cytometric analysis to identify major lymphocyte subpopulations in peripheral blood (Fig. 1B). This analysis revealed significantly (p<0.05) elevated levels of circulating CD3^+^ T cells at 60 days post-transplant as compared to baseline levels only in the allogeneic treatment group but no changes in CD4^+^ T cell counts were evident within or between treatment groups (Fig. 1C). Post-transplant levels of circulating CD20^+^ B cells, CD8^+^ T cells, and CD16^+^ NK cells were also elevated as compared to baseline levels in the allogeneic treatment group but these changes failed to achieve statistical significance (Fig. 1C). However, post-surgical CD16^+^ levels varied significantly [F(2,11) = 8.04, p = 0.01] between treatment groups.

### Allogeneic MSC Administration Induced Expansion of NKT and Major NK Subsets in Peripheral Blood

**Figure 2:**
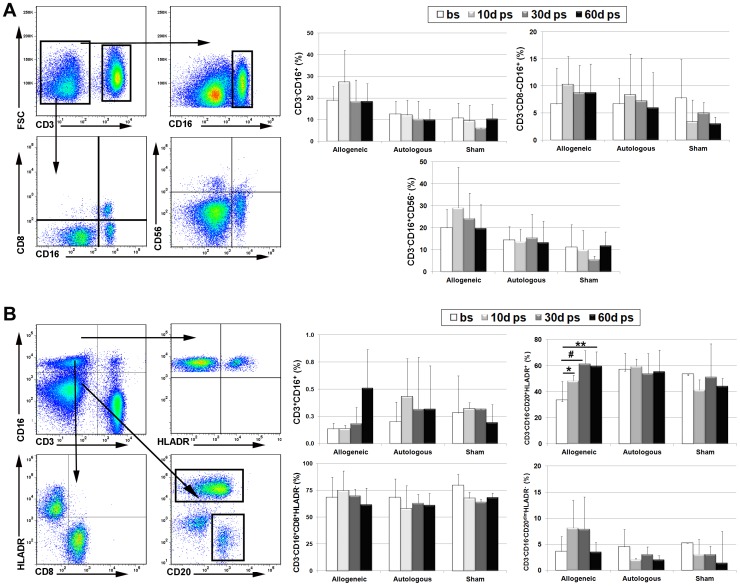
Allogeneic MSC administration induces expansion of circulating NK, NKT and B cell subsets in peripheral blood. (**A, B**) Representative density plots illustrating the gating strategy used to identify CD3^−^CD16^+^, CD3^−^CD8^−^CD16^+^ and CD3^−^CD16^+^CD56^−^ NK cell subsets (**A**) and CD3^+^CD16^+^ NKT, CD3^−^CD16^+^HLADR^+^ mDC, CD3^−^CD8^+^CD16^+^HLADR^-^ NK and CD3^−^CD16^−^CD20^+^HLADR^+^ B cell subsets (**B**) in peripheral blood by flow cytometry. Plotted values (mean ± SD) represent percentage of different lymphocyte subsets averaged for all transplant recipients within each respective treatment group at the indicated time points. Baseline values were determined as described in figure 1. * P<0.05, **P<0.01. P values were determined using unpaired, two-tailed Student t Test throughout.

To examine the host immune response in more detail, we next examined changes in natural killer (NK) cell subsets in peripheral blood. Rhesus macaque NK cell are defined as CD3^−^CD16^+^ or CD3^−^CD8^+^CD16^+^ [28], [29]. Gating on the CD3^−^ population identified a major subpopulation of CD16^+^ cells that transiently increased at 10 days post-transplant only in the allogeneic group. Moreover, CD3^−^CD16^+^ levels also varied significantly between treatment groups [F(2,11) = 15.39, p = 0.0012] (Fig. 2A). We also identified within the CD3^−^ population subsets of CD8^−^CD16^+^ and CD8^+^CD16^+^ cells (Fig. 2A). Post-transplant increases in CD3^−^CD8^−^CD16^+^ levels varied significantly between treatment groups [F(2,11) = 15.39, p = 0.0012] but no changes in CD3^−^CD8^+^CD16^+^ levels were evident within or between treatment groups (not shown). We also identified within the CD3^−^ population a small percentage of cells that expressed CD56 but this subset was poorly defined (Fig. 2A). This result is consistent with reports indicating that CD56^−^CD16^+^ cells are the dominant NK subset in peripheral blood and that CD56^+^ cells reside predominantly in lymph nodes [30]. Nevertheless, the CD3^−^CD16^+^CD56^−^ NK subset was elevated post-surgery only in the allogeneic treatment group and also varied significantly [F(2,11) = 20.28, p = 0.0005] between treatment groups (Fig. 2A). Pre-surgical baseline levels of all lymphocyte populations did not differ significantly between treatment groups.

The gating strategies employed above likely overestimated NK cell numbers in peripheral blood by including CD20^+^ B cells that also express CD8 and myeloid dendritic cells (mDC) that express CD16. Therefore, we used an alternative strategy to identify NKT, mDC, and NK subsets in peripheral blood [31]. For example, staining cells for CD3 and CD16 revealed a small subset of double positive NKT cells (Fig. 2B), which increased nearly 4-fold compared to baseline levels at 60 days post-transplant in the allogeneic treatment group. Gating on the CD3^−^CD16^+^ population also revealed a small subpopulation of CD16^+^HLADR^+^ mDCs and CD8^+^HLADR^-^ NK cells, which did not vary significantly within or between treatment groups (Fig. 2B). CD3^−^CD16^−^ cells were subdivided into CD20^+^HLADR^+^ B cells and a minor subpopulation of CD20^−/dim^HLADR^-^ NK cells (Fig. 2B). The B cell subset was significantly elevated (p<0.05) at all times post-transplant and levels of CD20^−/dim^HLADR^-^ NK cells also trended upward post-transplant in the allogeneic treatment group.

### Donor vs. Recipient Haplotype Influences the Magnitude of the Allo-immune Response

**Figure 3:**
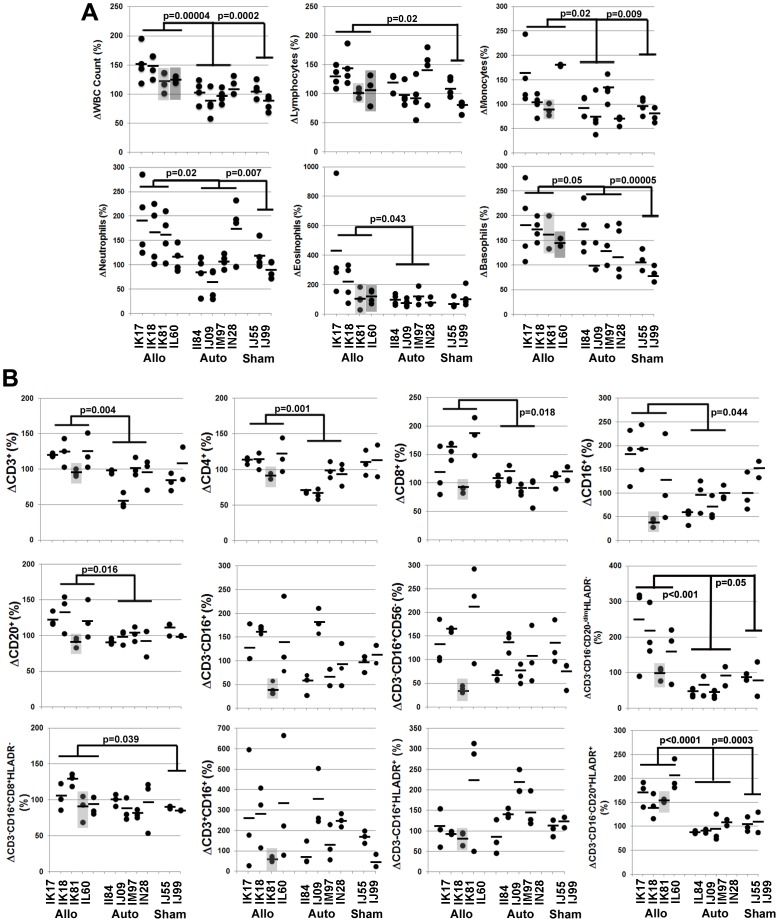
Degree of MHC mismatch between MSC donor and host dictates the magnitude and nature of the allo-graft response. (**A, B**) Complete blood cell counts (**A**) and circulating levels of lymphocyte subsets (**B**) quantified at 10, 30, 60 and 150 days post-transplant by automated counting and flow cytometry, respectively, were normalized to baseline levels for each transplant recipient. Plotted values represent the percent increase post-transplant at each time point and the horizontal bars represent the mean percent increase for each data set. Light and dark gray boxes depict data sets for allogeneic recipients IK80 and IL60, respectively. P values were determined using unpaired, two-tailed Student t Test throughout. Allo, allogeneic; Auto, autologous.

In most cases analysis of post-transplant lymphocyte counts failed to reveal significant differences between treatment groups due to the large variance among individual animals. However, closer inspection of this data indicated that this variance was largely contributed by data from a single animal. Therefore, we normalized post-surgical cell numbers to pre-surgical baseline levels for each animal, which revealed two important findings. First, this approached revealed that post-transplant increases in WBC, lymphocyte, monocyte, neutrophil, eosinophil, and basophil counts were all significantly (p<0.05) higher in the allogeneic vs. autologous and/or sham treatment groups (Fig. 3A). Second, post-surgical differences in complete blood cell counts varied to a lesser extent in allogeneic recipients IK81 and IL60, which matched their respective MSC donors at the Mamu A01 or DBRw201 alleles, respectively (Table 1).

**Table 1:** Donor and recipient MHC Class I and II antigen profile and allo-antibody status.

MSC Donor	Transplant Recipient	Mamu Allele Expression				Relative Allo-Reactivity
		A	B	E	DBR	
336-03		01	01,26	g1	w201	NA*
	IK17	07	19,24	1		+++
93-04		01,08	17,47	3	w201	NA*
	IK18	08,25	32,50	2		++
	IK81	01,08	01,29,74	2		−
	IL60	08	01,57	g1	w201	+/−

* Not analyzed.

Normalization of the flow cytometric data also demonstrated significant (p<0.05) differences in circulating CD3^+^, CD4^+^, CD8^+^, CD16^+^, and CD20^+^ subsets between the allogeneic vs. autologous treatment group (Fig. 3B). Once again, this analysis revealed that allogeneic recipient IK81 exhibited a weak allo-immune response, which is predicted based on haplotype (Table 1). Post-surgical changes in CD3^−^CD16^+^ and CD3^−^CD16^+^CD56^−^ NK subsets as well as NKT and mDCs were more variable and did not yield statistical differences between treatment groups. However, post-surgical increases in the CD3^−^CD16^−^CD20^−/dim^HLADR^-^ NK subset and CD3^−^CD16^−^CD20^+^HLADR^+^ B cells were significantly (p<0.05) greater in the allogeneic vs. autologous and sham treatment group and post-transplant increases in CD3^−^CD16^+^CD8^+^HLADR^-^ NK cells were also significantly higher in the allogeneic vs. sham treatment group (Fig. 3B).

### Secondary Antigenic Challenge Revealed Evidence of Allo-recognition by B Cells

**Figure 4:**
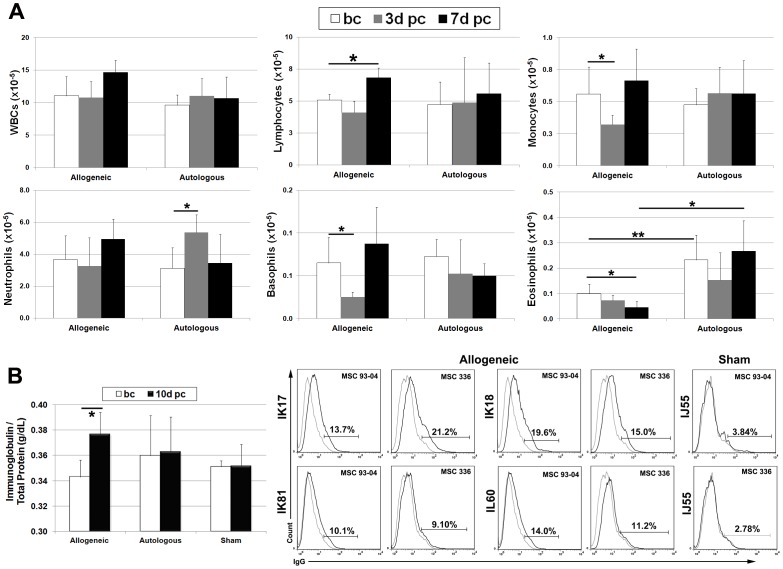
Secondary antigenic challenge reveals evidence of allo-antigen recognition by B cells. (**A**) Peripheral blood samples harvested at 1 month and 1 day prior to challenge (bc) and 3 and 7 days post-challenge (pc) were analyzed by automated counting to determine the number of WBCs, lymphocytes, monocytes, neutrophils, basophils and eosinophils. Pre-challenge data points were averaged to determine a new baseline for each transplant recipient. Plotted values (mean ± SD) represent cell counts averaged for all transplant recipients within each respective treatment group at the indicated time points. (**B**) The graph (left panel) illustrates sera immunoglobulin levels (mean ± SD) averaged for each treatment group before challenge (bc) and at 10 days post-challenge (pc). The right panel illustrates flow cytometric analysis of donor MSCs pre-incubated with sera of each indicated transplant recipient and stained with a FITC-conjugated antibody against the λ-chain of rhesus immuno-globulins (black line). Area under the gray line represents staining obtained with the isotype control. Both MSC donor populations (336-03 and 93-04) used for transplantation were evaluated. * P<0.05, **P<0.01. P values were determined using unpaired, two-tailed Student t Test throughout.

Next we challenged each transplant recipient with a subcutaneous injection of 1×10^6^ donor cells six months after initial exposure. To analyze the peripheral immune response, we sampled peripheral blood at 3–4 weeks and 1 day prior to challenge to establish a new baseline. Complete blood cell counts revealed a significant (p<0.05) increase in lymphocyte numbers at 7 days post-transplant as compared to baseline in the allogeneic treatment group (Fig. 4A). Allogeneic recipients also exhibited significantly (p<0.05) lower monocyte, basophil and eosinophil counts at 3 or 7 days post-challenge as compared to baseline levels. The only significant change detected in the autologous treatment group was an increase in neutrophil counts at 3 days post-transplant. However, we did not observe any significant difference in pre or post-challenge blood cell counts between treatment groups except for eosinophils, which were significantly higher in the autologous treatment group.

Quantification of sera immuno-globulin levels at 10 days post-challenge also revealed a significant (p<0.05) increase in allogeneic but not the autologous or sham treatment groups (Fig. 4B). Consistent with these findings, flow cytometry analysis of donor MSCs incubated with sera from each transplant recipient and stained with a rhesus-specific, FITC-conjugated antibody against the immunoglobulin λ-chain detected the presence of MSC-specific antibodies in allogeneic recipients (Fig. 4B). Allo-antibodies were cross-reactive with both rhesus donor MSC populations (93-04 and 336), which was consistent with the fact that they shared a Mamu A001/B047/DRBw201 haplotype. Sera from recipient IK81 consistently showed the lowest level of reactivity against donor MSCs, which reflected the weaker allo-immune response detected in this animal. Sera derived from sham-operated controls showed no reactivity against the donor MSCs.

### Secondary Antigen Challenge Increased mDC Levels in Peripheral Blood and NK Cells in Lymph Nodes of Allogeneic Recipients

**Figure 5:**
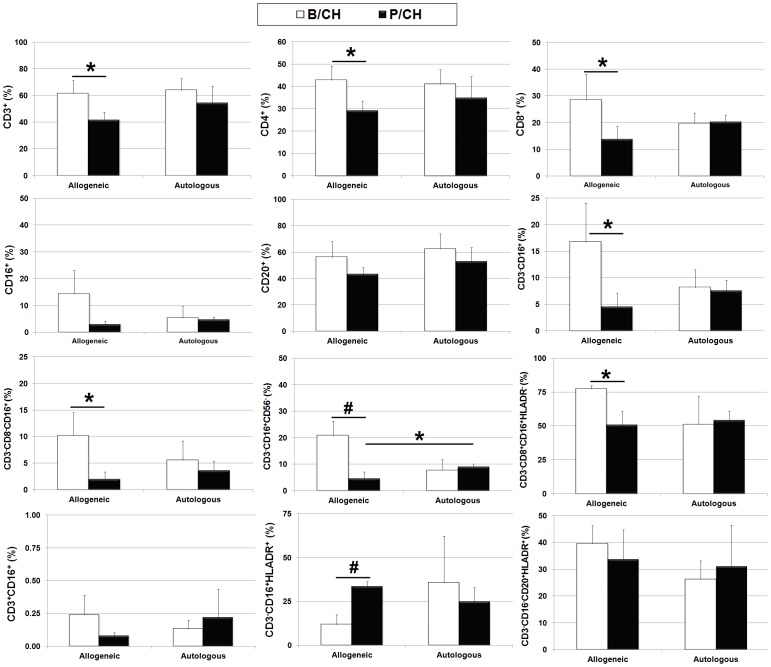
Secondary antigenic challenge increases mDC levels in allogeneic recipients. Plotted values (mean ± SD) represent average levels of circulating lymphocyte populations for the indicated treatment groups. Values were determined from peripheral blood before secondary antigen challenge (B/CH) and at 7 days post-challenge (P/CH). Pre-challenge baseline values were determined as described in figure 4. *P<0.05, **P<0.01. P values were determined using unpaired, two-tailed Student t Test throughout.

Flow cytometric analysis of peripheral blood samples revealed significant (p<0.05) decreases in circulating levels of CD3^+^, CD4^+^ and CD8^+^ subsets and all NK cell subsets at 7 days post-challenge as compared to baseline in the allogeneic treatment group. Post-transplant levels of CD16^+^ and CD20^+^ cells also were lower compared to baseline levels but these changes were not statistically significant (Fig. 5). However, we failed to detect any significant differences in post-challenge lymphocyte counts between the treatment groups except for CD3^−^CD16^+^CD56^−^ NK cells, which were significantly (p<0.05) higher in the autologous vs. allogeneic treatment group. Therefore, secondary antigenic challenge failed to induce a measurable immune response in allogeneic recipients apart from the detection of allo-specific antibodies. This is consistent with the fact that CD3^−^CD16^+^HLADR^+^ mDCs, which play a pivotal role in peripheral tolerance, were the only lymphocyte subset that was significantly elevated in the allogeneic treatment group post-challenge (Fig. 5).

**Figure 6:**
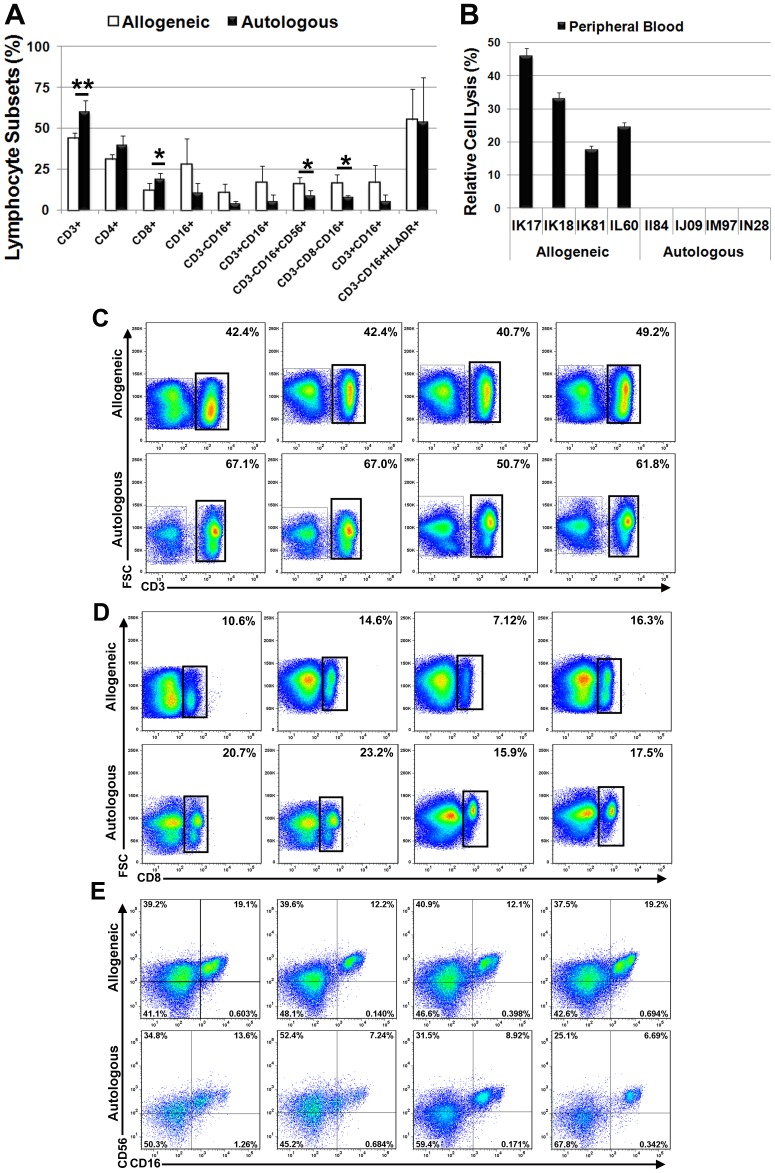
Elevated NK cells in lymph nodes of allogeneic recipients following secondary antigenic challenge. (**A**) Plotted values (mean ± SD) represent average levels of each lymphocyte sub population detected in lymph nodes by flow cytometry in the allogeneic or autologous treatment groups. * P<0.05, **P<0.01. P values were determined using unpaired, two-tailed Student t Test (**B**) Extent of cell lysis (mean ± SD) detected following a 4 h incubation of host PBMNCs with donor MSCs. (**C–E**) Density plots showing levels of CD3^+^ (**C**), CD8^+^ (**D**) and CD3^−^CD16^+^CD56^+^ (**E**) subsets detected in lymph nodes of each transplant recipient two week after secondary antigenic challenge. Allogeneic recipients - IK17, IK18, IK81 and IL60 (right to left). Autologous recipients – II84, IJ09, IM97, IN28 (right to left).

Analysis of lymph nodes harvested at two weeks post-challenge also revealed significant differences in lymphocyte counts between treatment groups. For example, lymph nodes from allogeneic recipients contained significantly lower levels of CD3^+^ and CD8^+^ T cells (Fig. 6A, 6C–D) and significantly greater numbers of CD3^−^CD8^−^CD16^+^ NK subset in the allogeneic vs. autologous treatment group (Fig. 6A). As predicted based on previous studies (34), we also detected a population of CD3^−^CD16^+^CD56^+^ NK cells in lymph nodes, which were also significantly elevated in the allogeneic vs. autologous treatment group (Fig. 6A, 6E). Finally, peripheral blood lymphocytes derived from allogeneic but not autologous transplant recipients exhibited the ability to lyse their respective donor MSCs in co-culture experiments *in vitro* (Fig. 6B).

### Cells Dose Inversely Correlates with MSC Engraftment Levels *in vivo*

**Figure 7:**
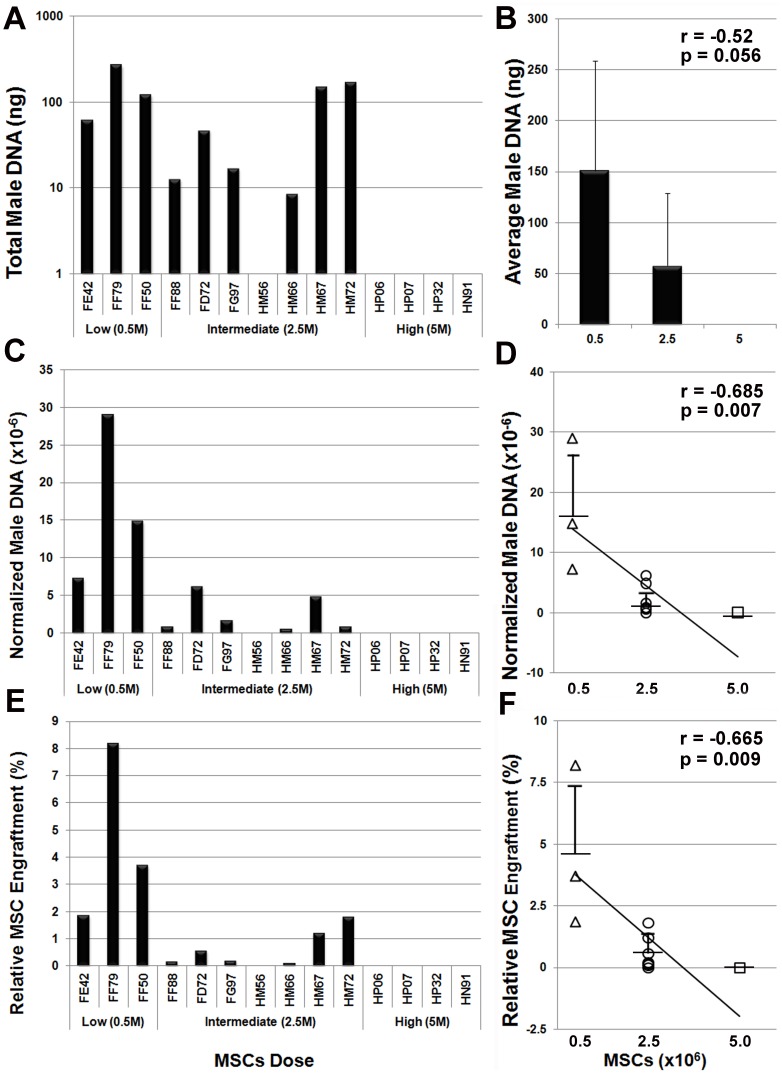
Cell dose-dependent effects on allogeneic MSC engraftment levels *in vivo*. (**A**) Plotted is total male DNA levels detected in all brain specimens of each transplant recipient analyzed by real-time PCR. (**B**) Plotted is the average (mean ±SD) male DNA content of brain tissue from all animals in each treatment group. (**C**) Plotted is the total amount of male DNA normalized to the total yield of genomic DNA from brain tissue from each transplant recipient. (**D**) Normalized male DNA levels (mean ± SD) within each treatment group were negatively correlated with cell dose (Pearson’s r = −0.685, p = 0.007). (**E**) Male DNA levels in brain were extrapolated to absolute cell numbers and used to calculate the percentage of injected MSCs that persisted *in vivo* at six months post-transplant. (**F**) Relative MSC engraftment levels (mean ± SD) within each treatment group were negatively correlated with cell dose (Pearson’s r = −0.665, p = 0.009).

Using a real-time PCR assay that targets sequences in the *Macaca sp.* SRY gene we quantified MSC engraftment levels in brain tissue from allogeneic recipients. We then pooled these data with that obtained previously from animals administered varying MSC doses to analyze effects of cell dose on MSC engraftment levels in brain [16], [24], [25]. This analysis revealed significant differences [F(2,11) = 4.02, p = 0.048] between treatment groups in overall male DNA levels recovered in brain tissue (Fig. 7A). However, male DNA levels were not significantly correlated with cell dose (Pearson’s r = −0.52, p = 0.056). In contrast, when normalized to total yield of genomic DNA per brain male DNA levels were also significantly [F(2,11) = 11.69, p = 0.0019] different between treatment groups and inversely correlated with cell dose (r = −0.686, p = 0.007) (Fig. 7B). Post-hoc comparisons revealed that normalized male DNA engraftment levels in the treatment group that received 0.5×10^6^ MSCs (mean = 17.01, SD = 11.02) were significantly different (α = 0.01) compared to treatment groups that received 2.5×10^6^ MSCs (mean = 2.12, SD = 2.39) or 5×10^6^ MSCs (mean = 0, SD = 0). However, mean values were not significantly different between treatment groups administered 2.5×10^6^ vs. 5×10^6^ MSCs (α = 0.05). Extrapolating male DNA levels to cell number further revealed that only a small percentage (0–8.2%) of injected MSCs were detected in brain tissue at 6 months post-transplant (Fig. 7C) but these values were also significantly different between treatment groups [F(2,11) = 9.77, p = 0.0036) and inversely correlated with cell dose (r = −0.666, p = 0.009) (Fig. 7C). In these studies, the cell volume administered intra-cranially was well below the maximum tolerated dose [25]. Therefore, it is unlikely that differences in MSC engraftment levels resulted from differences in the total number of injections administered per animal.

**Figure 8:**
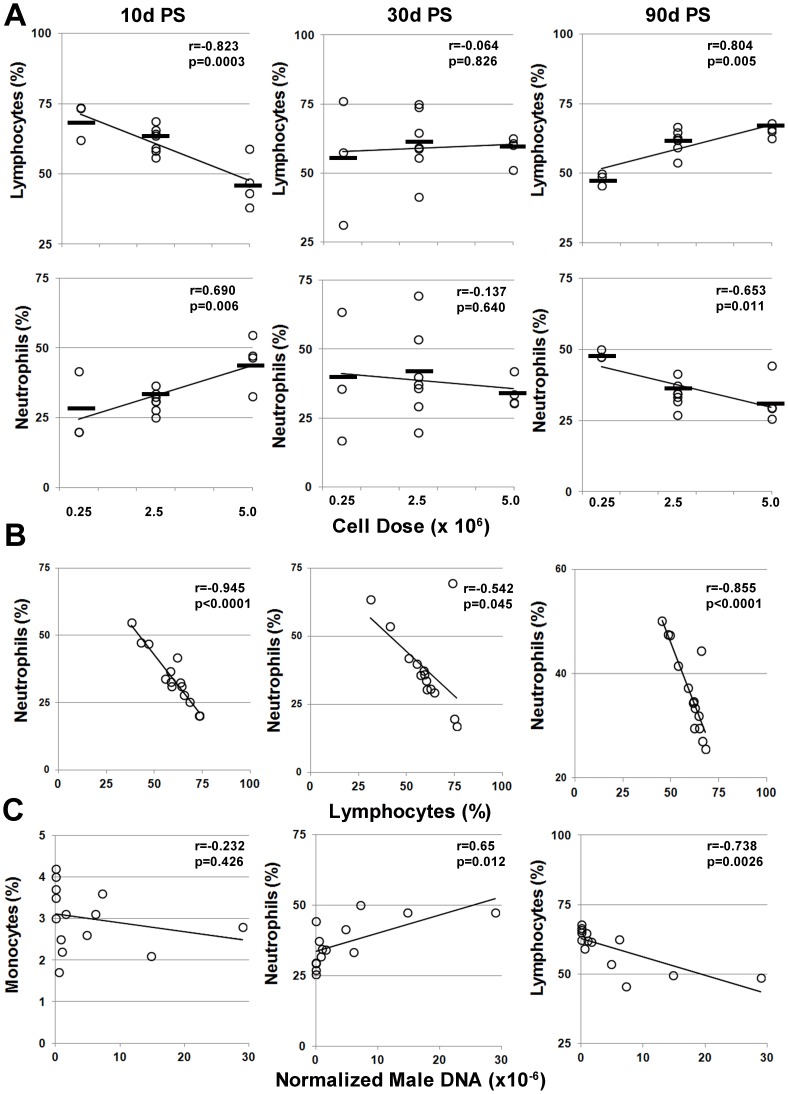
Post-transplant changes in circulating neutrophil and lymphocyte counts correlate with MSCs dose and engraftment levels. (**A**) Absolute lymphocyte and neutrophil counts (%) in peripheral blood at 10, 30, and 90 days post-transplant were plotted as a function of cell dose for each transplant recipient. Bars represent mean cell count for each treatment group. (**B**) Correlation between neutrophil and lymphocyte counts from (**A**). (**C**) Absolute lymphocyte and neutrophil counts (%) in peripheral blood plotted as a function of normalized male DNA levels in brain tissue from all transplant recipients. Abbreviations: r, the Pearson’s product- moment correlation coefficient; p, corresponding p value.

As shown in Figure 8A, post-transplant lymphocyte counts negatively correlated with cell dose at 10 days post-transplant (r = −0.823, p = 0.0003), showed no correlation at 30 days post-transplant (r = 0.065, p = 0.862) and correlated with cell dose at 90 days post-transplant (0.804, p = 0.0005). Lymphocyte counts were also inversely correlated (r = −0.738, p = 0.003) with MSC engraftment levels in brain at 90 days post-transplant (not shown). In contrast, post-transplant neutrophil counts correlated with cell dose at 10 days post-transplant (r = 0.692, p = 0.006), showed no correlation at 30 days post-transplant (r = −0.137, p = 0.64) and inversely correlated with cell dose at 90 days post-transplant (r = −0.653, p = 0.011) (Fig. 8A). As anticipated based on these results, neutrophil and lymphocyte counts were inversely correlated at all time points post-transplant (Fig. 8B). Moreover, neutrophil and lymphocytes counts positively and inversely correlated with MSC engraftment levels, respectively, while monocyte counts shown no correlation (Fig. 8C).

### Affect of MSC Administration on Animal Behavior

**Figure 9:**
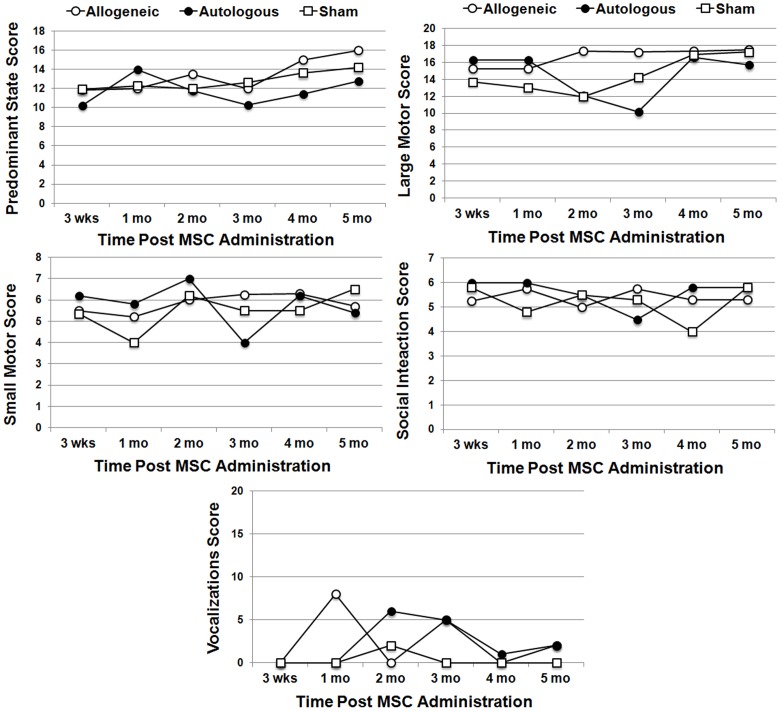
Effect of allogeneic and autologous MSC administration on animal behavior. Plotted are outcomes from socialization/exercise cage (or ‘play cage’) evaluations conducted over the 6 month study period. Test scores were groups into four categories for analysis including large motor, small motor, social, and behavioral state. Data were analyzed using repeated measures ANOVA, which revealed a significant difference [F(5,20) = 5.67, p<0.005] in large motor movements between the allogeneic vs. autologous treatment group.

We also evaluated the behavior of transplant recipients using age and species appropriate tests throughout the time course of the experiment. Neurobehavioral assessment scores for all 10 macaques recruited into this study fell within two standard deviations of the mean calculated for a normative control group, confirming their neural development was normal prior to the start of the study (not shown). Animals were then administered a battery of age-appropriate tests to evaluate motor skills, social development, and temperament. No significant differences were noted over the six-month time course in predominant state, small motor function, social interaction or vocalization between treatment groups (Fig. 9). However, there was a significant difference [F(5,20) = 5.67, p<0.005] in large motor movements between the allogeneic vs. autologous treatment group. Herein, scores were higher at two and three months post-transplant in the allogeneic recipients but this difference was no longer evident at 4 months post-transplant.

We also compared effects of MSC dose on animal behavior across all animal cohorts administered allogeneic MSCs. Herein, cell dose did not correlate with post-surgical Bayley assessments, which measured problem solving ability [F(2,11) = 12.742, p = 0.108], motor maturity [F(2,11) = 1.68, p = 0.23] and social behavior [F(2,11) = 0.171, p = 0.845]. Moreover, there was no effect of cell dose on large motor abilities (F(3,12) = .731, p = .553), small motor abilities (F(3,12) = .1.22, p = .346), social behavior (F(3,12) = 1.80, p = .201), or behavioral state (F(3,12) = 1.93, p = .179) and inclusion of sham subjects into the model did not alter these findings. Therefore, despite significant differences in overall MSC engraftment levels between treatment groups, no long-term effects of MSC administration on animal behavior was detected.

## Discussion

**Figure 10:**
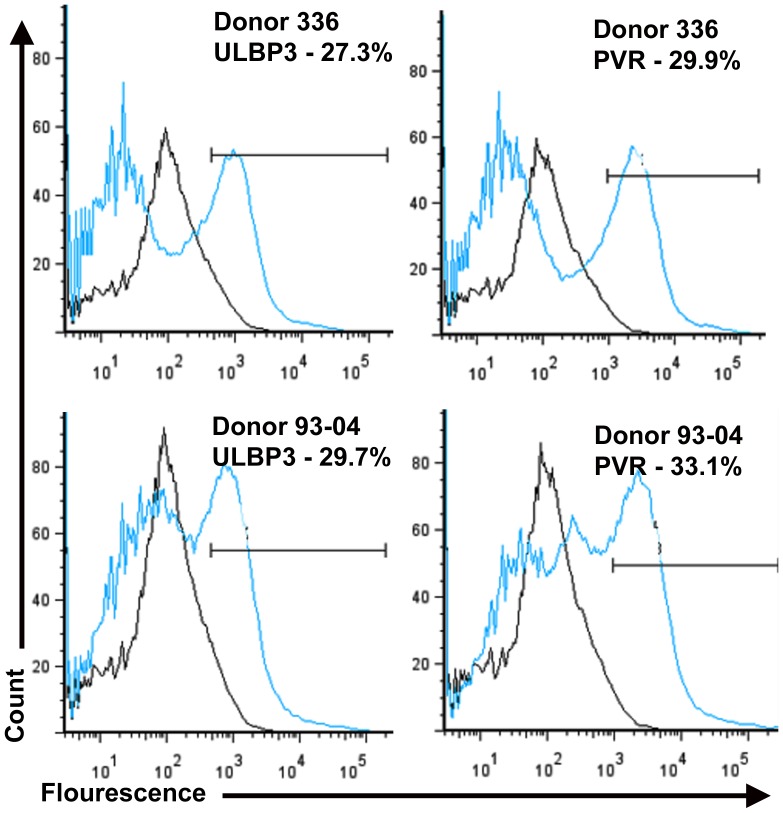
MSC donor populations express ligands that induce NK receptor activation. Flow cytometric analysis of MSC donor populations 93-04 and 336-03 stained with an isotype control (gray line) or antibodies specific for ULBP3 and PVR (blue line). Indicated is the percentage of cells immuno-reactive against each respective antibody.

Several human clinical trials are currently evaluating allogeneic MSCs [32], [33] based on evidence that these cells do not exhibit acute immune-mediated toxicity following infusion into unrelated human patients. However, lack of infusion-related toxicity in humans does not preclude allo-reactivity, a phenomenon frequently observed in experimental animal models following infusion of allogeneic MSCs [10]–[16]. Data from this study provide unambiguous evidence of productive allo-recognition by B cells and anti-donor T and NK cell responses following allogeneic MSC administration into immuno-competent rhesus macaques, which are consistent with previous findings [16]. However, by directly comparing allogeneic vs. autologous MSCs expanded under identical conditions the current study rules out artifacts produced by contamination of culture expanded MSCs with xeno-antigens and/or viruses. In addition, the degree of MHC class I and II mismatch between donor and host influenced the magnitude and nature of the host allo-response. For example, recipients IK17 and IK18 exhibited increases in neutrophil, basophil, and eosinophil counts as well as NK and B cell subsets in peripheral blood, which also contained detectable allo-specific antibodies, indicative of a Th2-mediated allo-immune response. This result is consistent with studies indicating that neutrophils produce cytokines that modulate B, T and dendritic cell function [34], [35] and basophils induce B cell proliferation and class switching via secretion of IL4 and IL13 and expression of CD40 ligand [36]. Moreover, eosinophils also reportedly participate in graft rejection particularly in the absence of a CD4 T cell mediated Th1 response [37]. Expansion of NK subsets in these recipients is also consistent with mismatches at the Mamu E allele and expression by donor MSCs of the poliovirus receptor (PVR) and UL16 binding protein 3 (ULBP3) (Fig. 10), which bind to activating receptors on NK cells [38], [39]. Indeed, MSCs are subject to lysis by NK cells [40] and rapid NK cell expansion is also seen in patients receiving allogeneic hematopoietic stem cell transplants [41]. These responses were largely absent from recipient IK81, which was matched at the major Mamu A allele with its respective MSC donor. Alternatively, recipient IL60 exhibited the largest expansion of CD3, CD4 and CD8 T cells, modest expansion of basophils or eosinophils, and lower levels of allo-specific antibodies, which is indicative of a Th1-mediated response. This result is consistent with the fact that IL60 was partially matched at Mamu A and DBR alleles and expressed Mamu B alleles that are phylogenetically related to the respective MSC donor [42].

Analysis of allogeneic MSC engraftment levels in brain tissue at 6 months post-transplant revealed an inverse correlation with cell dose. Post-transplant changes in circulating neutrophil and lymphocyte counts also exhibited time dependent correlations with cell dose and MSC engraftment levels. These findings indicate that host allo-graft responses limit allogeneic MSC engraftment *in vivo*, and are consistent with numerous studies implicating increased neutrophil infiltration at early stages of allograft rejection [43]–[45] with augmented T cell mediated allo-immunity [46], [47]. Moreover, our data are consistent with a recent study by Kirsner et al. [48] showing that lower MSC doses are more effective for the treatment of chronic venous leg ulcers in human patients. Indeed, even if MSCs function by a “hit and run” mechanism it is anticipated that cell dose will significantly influence therapeutic outcome especially in an allogeneic transplant setting. For example, studies have demonstrated a lack of long-term efficacy of MSC-based therapies in patients with rheumatoid arthritis [49] and/or reduced benefit of allogeneic cells in animal models due to rejection by the host immune system [50]. Moreover, administration of autologous vs. allogeneic MSCs in cardiac patients was associated with an improvement in the 6-minute walk test, the Minnesota Living with Heart Failure Questionnaire score and a larger decrease in infarct size [33]. Therefore, durable long-term engraftment may be necessary to produce sustained therapeutic benefits in a subset of diseases.

In our study secondary antigenic challenge failed to induce a robust secondary immune response in allogeneic recipients, which is consistent with a previous report in baboons demonstrating reduced anti-donor T cell reactivity following challenge with a second inoculum of allogeneic MSCs [15]. This outcome is also reminiscent of outcomes seen following repeated administration of immature allogeneic DCs in humans [51]. Indeed, mDCs were the only lymphocyte subset induced in allogeneic recipients following secondary antigen challenge and may contribute to the lack of allo-reactivity following secondary challenge.

In this study MSCs were administered intra-cranially. This delivery route is significant in that the CNS has no direct lymphatic drainage and is protected by a blood-brain barrier [52]. Consequently, the proportion of T lymphoblasts that gain entry to the CNS is lower than seen in other organs [53] although microglia and peri-vascular macrophages resident in the brain also function in antigen presentation, inflammatory processes, and removal of apoptotic cells and cellular debris [54]. Therefore, the host immune response may differ following intracranial vs. systemic MSC administration, whereas the latter route is anticipated to produce a more robust allo-graft reaction. While our study failed to detect any effects of cell dose on animal behavior and motor function, we cannot rule out the possibility that immunological rejection of allogeneic MSCs induces localized tissue inflammation and cell death. Therefore, metrics to evaluate host allo-reactivity and its effects on treatment efficacy and patient health are a necessary component to any allogeneic MSC-based clinical therapy.
